# Composition and Nutrient Information of Non-Alcoholic Beverages in the Spanish Market: An Update

**DOI:** 10.3390/nu8100618

**Published:** 2016-10-08

**Authors:** María Serrano Iglesias, María de Lourdes Samaniego Vaesken, Gregorio Varela Moreiras

**Affiliations:** Department of Pharmaceutical and Health Sciences, Faculty of Pharmacy, CEU San Pablo University of Madrid, Madrid 28668, Spain; maria.serranoiglesias@ceu.es (M.S.I.); l.samaniego@ceu.es (M.d.L.S.V.)

**Keywords:** non-alcoholic beverages, Spanish market, food composition database

## Abstract

The aim of this study was to draw an updated map of the nutrition facts in the different categories of non-alcoholic beverages in the Spanish market based on the information declared on the labels of these products; we expect this first step to justify the need for the coordination and harmonization of food composition tables in Spain so that there will be an updated database available to produce realistic scientific nutrient intake estimates in accordance with the actual market scenario. Materials and Methods: The nutrition facts declared on the labels of non-alcoholic beverages by manufacturers in Spain were compiled and studied. Results: The database included 211 beverages classified in 7 groups with energy/carbohydrate content per 100 mL ranging from 0–55 kcal/0–13 g for soft drinks; 2–60 kcal/0–14.5 g for energy drinks; 24–31 kcal/5.8–7.5 g for sports drinks; 1–32 kcal/0–7.3 g for drinks containing mineral salts in their composition; 14–69 kcal/2.6–17 g for fruit juice, nectar, and grape musts; 43–78 kcal/6.1–14.4 g for vegetable drinks; and 33–88 kcal/3.6–14 g for dairy drinks. Conclusion: The current non-alcoholic beverage market is a dynamic, growing, and highly innovative one, allowing consumers to choose according to their preferences, needs, or level of physical activity at any moment of the day.

## 1. Introduction

Food Composition Tables (FCTs) and Databases (FCDB) are essential tools to help nutritionists and dieticians in designing and assessing diets, through the knowledge of the composition and nutritional value of foods. Nowadays, several FCDBs from different institutions and research centers are available at an international level, the EuroFIR project [1] (European Food Information Resource Network of Excellence) highly contributed by establishing a European database platform that uses standardized criteria for compiling and managing food composition data. There are other databases such as the widely used US Department of Agriculture (USDA) FCDB [2]. At present, most countries have developed and published their own FCT or FCBD, and a comprehensive inventory can be found at FAO’s INFOODS International Network webpage (http://www.fao.org/infoods/infoods/tables-and-databases/en/) [3]. In Spain, the database of food composition, developed by the Spanish Food Composition Database (BEDCA network) [4] in cooperation with the Spanish Agency for Consumer Affairs, Food Safety and Nutrition (AECOSAN), was built upon the European standards developed by EuroFIR [1] and the LanguaL™ [5] food description system, all of which aimed at harmonizing FCDB across Europe. Other examples include the FCT by Moreiras et al. [6], the one designed by the Nutrition and Dietetics Higher Education Centre of Barcelona (CESNID) [7], the ones by Mataix [8]—which were compiled by researchers from the Institute of Nutrition and Food Technology of the University of Granada—and the ones by Ortega et al. [9] from which the “DIAL” nutrition assessment software was designed. However, preparing, maintaining, and updating FCTs and FCBDs is a complex process. In Spain, each one of the aforementioned tables or databases have been developed following different methodologies and by including a number of data sources: Analytic data from research teams, data from Spanish and foreign FCT, scientific publications, data from the agro-food industry, and extrapolations or estimates. Therefore, food nomenclature and technological and culinary processes do not normally follow a single standard [10,11], and this is considered a main limitation. In addition, FCTs and FCBDs are generally difficult to update as the market rapidly evolves due to the great variety of available food and beverage products. From all these considerations, it can be inferred that there are a number of limitations and deficiencies to be overcome when it comes to food composition availability.

The non-alcoholic drinks group is nowadays the one with the highest consumption in the global Spanish diet; it also presents the fastest evolution in the last decades and the highest levels of innovation in many different aspects (i.e., reduced calorie content; flavor) [12,13]. According to the Spanish Food Consumption Panel of 2014, the soft drinks group is the one with the highest consumption per capita, with 42 L/person/year followed by the category of juices and nectars (10 L/person/year) [14]. Consumers can choose from a wide range of sparkling and still beverages, sugar or sugar-free, fat or non-fat, with or without fruit juice, vitamins, as well as different flavors, packaging materials, and sizes. All of this variety, however, is not readily reflected in the present in the Spanish FCT and FCDB.

Given the significant contribution of non-alcoholic beverages to diet and the lack of harmonized, comprehensive composition data, the present study aims to draw an updated map with the nutritional facts of the different categories available in the Spanish market based mainly on the data declared on the labels of these products. This tool should fulfill the limited information found in current FCTs; it will also offer an accurate update of those tables and reveal the most recent innovations in the Spanish food market. Secondly, the goal of creating a single database validated by EuroFIR guidelines [1] with periodic updates will allow for the production of sound energy and nutrient intakes estimates from non-alcoholic beverage consumption data in accordance with the actual market scenario and the consumption habits of the population.

## 2. Materials and Methods

### 2.1. Compilation of Food Composition Data for Non-Alcoholic Beverages

A large market research was undertaken at main retailers located in the Madrid Region that is representative of the Spanish market. Seven hypermarkets chosen according to their sales volumes were visited by one researcher who collected the data on site. For each visit, a work scheme aimed at covering the whole marketing surface dedicated to non-alcoholic beverages with manufacturer brand was followed: Soft drinks; energy drinks; sports drinks; drinks containing mineral salts among their ingredients; fruit juice, nectar, and grape musts; vegetable drinks; and dairy drinks. The LanguaL™ [5] food description and classification system was used, and beverages were classified into groups based on their energy density: “zero-calorie” beverages for those with energy density equal to or less than 4 kcal/100 mL and “low-calorie” drinks for those with energy density equal to or less than 20 kcal/100 mL [15]. After visiting retailers, the collected beverage inventory was double-checked for completeness by an on-line search comprising brand and retailer websites. Concerning nutrition facts, Regulation 1169/2011 on food information to consumers [16], which made the inclusion of the nutrition facts on the labels of food and drinks compulsory as from 13 December 2016, was used as criteria. Beverages label must clearly show, in an easy-to-read manner, the following information: Energy value, amount of fat, saturated fatty acids, carbohydrates, sugars, proteins, and salt. These contents may include the following substances: Monounsaturated fatty acids, polyunsaturated fatty acids, polyols, starch, dietary fiber, and any relevant quantities of vitamins or minerals (accounting for 7.5% of the daily reference intake). Finally, nutrient quantities are expressed in grams (g) per 100 mL, and energy value is expressed in kilojoules (kJ) and kilocalories (kcal) per 100 mL of drink. Taking into account all of the previous considerations, we compiled the compulsory nutrition facts from labels: Energy value, fat, saturated fats, carbohydrates, sugars, proteins, and salt. In addition, vitamins and minerals were compiled when declared.

### 2.2. Review of Spanish Food Composition Tables and Databases

We also conducted a review of the Food Composition Tables (FCTs) and Databases (FCDB) published at a national level in their latest editions (2004–2015). With the aim of compiling the nutrition facts of non-alcoholic beverages in the Spanish market and analyzing their classification, we used the four main FCTs [6,7,8,9] and the Spanish database for food composition (BEDCA) [4]. For each FCT and FCDB, we recorded their classification scheme and beverage composition data for further analysis and comparison.

### 2.3. Data Management

Data was compiled and recorded in Excel^®^ spreadsheets. The statistical data management was carried using the statistical analysis suite SPSS 18.0 (SPSS Inc., Chicago, IL, USA). For the obtained results, descriptive data were expressed as a range (minimum-maximum).

## 3. Results

### 3.1. Food Composition Database for Non-Alcoholic Beverages in the Spanish Market

A total of 211 non-alcoholic beverages were included and classified into seven groups including declared data on energy, fat, saturated fats, carbohydrates, sugars, proteins, and salt content per 100 mL. Table 1 shows a summary of global results for energy, carbohydrates, simple sugars, fats, and protein contents, of which the first three are the main contributions from these products. Table 2, Table 3, Table 4, Table 5, Table 6, Table 7 and Table 8 include detailed results of the different groups or categories included for the classification proposal.

Energy content per 100 mL ranged from 0 to 55 kcal for soft drinks as shown in Table 2; 2–60 kcal for energy drinks (Table 3); 24–31 kcal for sports drinks (Table 4); 1–32 kcal for drinks containing mineral salts in their composition (Table 5); 14–69 kcal for fruit juice, nectar, and grape musts (Table 6); 43–78 kcal for vegetable drinks (Table 7); and 33–88 kcal for dairy drinks (Table 8).

In the case of carbohydrates, the major component for this type of drinks, values per 100 mL ranged from 0 to 13 g for soft drinks (Table 2); 0–14.5 g for energy drinks (Table 3); 5.8–7.5 g for sports drinks (Table 4); 0–7.3 g for drinks containing mineral salts in their composition (Table 5); 2.6–17 g for fruit juice, nectar, and grape musts (Table 6); 6.1–14.4 g for vegetable drinks (Table 7); and 3.6–14 g for dairy drinks (Table 8).

Although the basic component of non-alcoholic beverages is water, it is not a compulsory required nutrient to be declared on labels, therefore it is rarely present in FCDBs or FCTs. It is followed in proportion by carbohydrates (2.6–14.5 g per 100 mL); however, today’s market reveals an important diversification reflecting the current demand of new products. To illustrate this trend, a 19% reduction in calories per liter took place for the global of soft drinks in the Spanish market as a consequence of consumer demand in the period between 2009 and 2015 [12]. Consumers can now choose carbonated or non-carbonated drinks, sugar or sugar-free, drinks containing fat or no fat (and also different fat profiles), drinks with or without fruit juice, drinks with vitamins, varied flavors, and packaging of different sizes and materials. In other words, the processes of product design, innovation, and reformulation are nowadays oriented towards very distinct “targets”. The analysis of ingredients in the case of soft drinks reveals that water can be carbonated or not. A syrup, or basic preparation from a mixture of different ingredients such as sugar, fruit juices, and aromas, is added to water for flavoring. Moreover, depending on the type of soft drink, the beverages may also contain caffeine, quinine, vitamins, minerals and no-calorie sweeteners. In this sector, innovation is mainly related to flavors; thus, every year new flavors are launched into the market. Although the most relevant percentages of production and consumption still remain the classic cola or citric flavors, new flavors are growing at faster rates [17]. Water accounts for about 90% of the total weight in fruit juices. Soluble sugars follow water in quantity; the most common are fructose, glucose, and sucrose. Fruits also contain dietary fiber, mainly as pectin. After sugar, organic acids are the second component in quantity, mainly citric acid and malic acid. Additionally, these may be enriched with vitamins [18]. Fruit nectar is the product obtained by the addition of water and sugar or honey to fruit juice. This addition is allowed in a quantity not greater than 20% of the total weight of the end product. At present, we can find in the market low calorie nectars that are produced by replacing soluble solids by sweeteners like aspartame or sucralose. Composition of different types of yogurts varies depending on the composition of the source milk, the quantities of added milk powder, and the strain and fermentation conditions. Further, edible sugars, authorized sweeteners, flavorings, juices, fruits, or other natural products can be added to the base product, resulting in the large variability in products within the sector [18]. Vegetable drinks are made from the juice of a vegetable source, to which sugar or sweeteners, salt, thickeners, and, in some cases, cocoa or other juices are added. They can also be enriched with vitamins and minerals. Following the analysis of nutrition facts in non-alcoholic beverages from the Spanish FCT, we observed that the results are not in agreement with the reality of the market, as they do not match the values declared in the nutrition facts labels by the manufacturers. Furthermore, most databases are based on the data found in the associated literature and from other databases; there are also attributed values and estimates, and the data do not usually include those new products now available for consumers. This combined method for data collection may be more cost-efficient, but it does not represent the current offer on the market. Therefore, attributed values and estimations should be kept to a minimum if we want to improve the reliability of the database [19].

### 3.2. Classification of Non-Alcoholic Beverages in the Spanish Food Composition Tables

Important differences were observed in the last editions of the FCT and FCBD concerning beverage classification (Table 9): The BEDCA [4] database and the tables by Moreiras et al. [6] classify them into two groups and no subgroups are defined. BEDCA [4] includes liquid yogurts and milkshakes into the dairy drinks and milk derivatives group, including drinks of vegetable origins, whereas non-dairy drinks include soft drinks, juices, nectars, and “horchatas” (“tiger nut milk”, typical of Spain). It is noteworthy that this database is the only one in line with the EuroFIR standards [2] and the LanguaL™ [5] classification criteria. The tables by Moreiras [6] include only liquid yogurts and milkshakes in this group, while vegetable drinks are included into the non-alcoholic beverages group, together with soya milkshakes, soft drinks, horchatas, and juices and nectars. The tables by Ortega [9] and CESNID [7] also define two groups, and a subgroup is distinguished for each one of them for the classification of beverages. The group of dairy drinks and milk derivatives includes milkshakes and liquid yogurts; the group of non-alcoholic beverages includes soft drinks, juices and nectars, and vegetable drinks. The Mataix [8] FCT define a three-group classification: fruit juices, milk and milk derivatives, and drinks in general. Therefore, clearly, the different and most used FCT do not follow a common set of classification criteria. As an example, vegetable drinks are sometimes considered dairy drinks but are also included in the general group of drinks; in the case of soft drinks, no subgroup is defined, even though it is a very wide and continuously growing category.

## 4. Discussion

In the present study, we intended to develop an updated tool comprising all available composition data for non-alcoholic beverages in the Spanish market. The need for updated and harmonized data is essential, as the Spanish FCT and FCDB do not accurately reflect the great variability found in the market. The newly created database should serve as a starting point for developing a unified FCDB that will include a growing offer of reformulated and new beverages; furthermore, this database should allow periodical updates and be a tool for sound dietary intake estimates in line with the reality of the market. Concerning the classification of beverages in the FCT, there is not a common set of classification criteria. In consequence, vegetable drinks are sometimes considered as dairy drinks and sometimes they are included in the general group of beverages. Moreover, and very significantly, there is not a subgroup for soft drinks, even though this is a very wide category. The use of different tables and databases in Spain has been a handicap when comparing dietary energy and nutrient data on previous studies [20]. Some recent examples of these dietary and nutritional studies carried out in Spain, which gathered information on the population for this purpose, are “ANIBES Study” [21], “ENIDE Survey” [22], and “ENPE Survey” [23]. Notwithstanding the efforts carried out in the last decade on the harmonization of food descriptions, nutrients terminology, analytic methods, and estimation and compilation methods, there is no doubt among the scientific community and those in charge of food and nutrition policies of the existence of a great variability in the results from FCT and FCDBs. We should therefore consider the discussion of the methods applied to convert food and beverages intake into nutrients, admitting they present advantages, inconveniences, biases, and limitations [24]. It must be accepted, however, that there is no gold-standard methodology—even more so when considering that available food products are continually increasing and being reformulated but still quite unknown in respect to their composition, or the synergies and antagonisms between their ingredients. Moreover, the different ways of processing, distributing, and conservating are also worth analyzing. One of the main reasons for keeping an updated database is the growing offer of new and reformulated food products in a short period of time. Reliable food composition data are necessary to assess and estimate energy and nutrient intake, as well as for the formulation of diets and the development of dietary recommendations [11]; it is therefore crucial to reach a wide consensus for the development of a database at a national level with high quality standards, which should be validated and accepted by international bodies [10]. The established protocols for compiling and imputing food composition data used in this work represent a valid methodology, but undoubtedly rely on the provision of sufficient resources for maintaining yearly updates of the FCBD. In this regard, governmental, scientific bodies and food/beverage industry interests and funding are deemed essential.

## 5. Conclusions

This compilation and update of non-alcoholic beverages nutrition facts from label data and new classification criteria in groups and subgroups is an easy-to-use and accurate tool for conducting dietary surveys and assessing the nutritional status among the Spanish population. There is a high number of non-alcoholic beverages in the Spanish market, which is a dynamic and a very innovative one, allowing consumers to choose according to their personal tastes, needs, and activities at any moment of the day. Creating an updatable database at a national level will not only allow for sound estimates on food intake with scientific validity but will also be in agreement with observed market innovations. Such a database should consider the following requirements: Ease of use, clear food descriptions, representative data of the country they belong to, good analytic quality, the coherent expression of data, methodological transparency, and the wide food and nutrient spectrum.

## Figures and Tables

**Table 1:** Energy and macronutrients content (range) per 100 mL in non-alcoholic beverages groups from the Spanish market.

	Energy (kcal)	Carbohydrates (g)	Simple Sugars (g)	Fats (g)	Protein (g)
**Soft drinks (*n* = 94)**	0–55	0–13	0–13	0–0.1	0–0.5
**Energy drinks (*n* = 12)**	2.2–60	0–14.5	0–14.5	0–0.5	0–1.3
**Sports drinks (*n* = 7)**	24–31	5.8–7.5	3.9–7.5	0	0
**Drinks containing mineral salts (*n* = 4)**	1–32	0–7.9	0–7.9	0	0
**Fruit juice, nectar, and grape musts (*n* = 49)**	14–69	2.6–17	2.6–17	0–0.5	0–0.8
**Vegetable drinks (*n* = 15)**	43–78	6.1–14.4	5.8–12.6	0–2.3	0.4–3.4
**Dairy drinks (*n* = 30)**	33–88	3.6–14	3.5–14	0.8–4	2.1–3.3

**Table 2:** Nutrition facts from the “soft drinks” group in the Spanish food market.

Beverages Group	Subgroups	*n*	Energy (kcal)	Fat (g)	SFA (g)	Carbohydrates (g)	Total Sugars (g)	Proteins (g)	Salt (g)
**Soft drinks**	COLA SOFT DRINK	2	42–43	0	0	10.6–10.7	10.6	0	0–<0.01
	COLA SOFT DRINK, CAFFEINE FREE	2	44	0	0	11.1	11.1	0	0–<0.01
	COLA SOFT DRINK, NO-CALORIE	4	0.2–0.5	0	0	0–0.1	0	0–0.05	<0.01–0.02
	COLA SOFT DRINK, NO-CALORIE, CAFFEINE FREE	4	0.2	0	0	0–0.05	0	0–0.05	<0.01–0.02
	COLA SOFT DRINK LEMON	1	42	0	0	10.6	10.5	0	0.03
	COLA SOFT DRINK, LEMON, NO-CALORIE	2	0.3–1	0	0	0	0	0–<0.1	0.02–0.03
	COLA SOFT DRINK CHERRY	1	43	0	0	10.7	10.7	0	0
	LEMON SOFT DRINK	6	27–43	0	0	6.2–10.5	6–10.5	0	0–0.06
	LEMON SOFT DRINK, NO-CALORIE	3	0.5–2	0	0	0–0.4	0–0.4	0	0–0.06
	LEMON SOFT DRINK—STILL	6	26–55	0	0	6.3–13	6.2–13	0	0–<0.1
	LEMON SOFT DRINK—STILL, NO-CALORIE	2	3	0	0	0.1–0.2	0.1	0.1	<0.01–0.05
	LEMON SOFT DRINK—STILL, LOW CALORIE	1	10	0	0	1.9	1.8	0	0.1
	LEMON LIME SOFT DRINK	2	44–49	0	0	11–12.3	11–12.2	0	0.02–0.04
	LEMON LIME SOFT DRINK, LOW CALORIE	1	10	0	-	2.1	2.1	0	0.02
	LEMON LIME SOFT DRINK, NO-CALORIE	2	0.1–1	0	0	0	0	0–<0.1	0.03–0.06
	ORANGE SOFT DRINK	4	29–52	0	0	6.9–12.7	6.7–12.7	0	0–0.03
	ORANGE SOFT DRINK, NO-CALORIE	2	4	0	0	0.9	0.9	0	0–0.02
	ORANGE SOFT DRINK—STILL	2	38	0	0	9.1–9.3	8.9–9.3	0–0.1	0–0.03
	ORANGE SOFT DRINK—LOW CALORIE CONTENT	5	6–17	0–0.1	0	0.8–4.1	0.8–4	0–0.1	<0.01–0.13
	STRAWBERRY SOFT DRINK	2	44–51	0	0	10.8–12.4	10.8–12.3	0	0
	PINEAPPLE SOFT DRINK	1	41	0	0	10.2	10.2	0.1	0.01
	APPLE SOFT DRINK	1	32	0	0	8	8	0	0
	CITRUS FLAVOURED SOFT DRINK	2	24–38	0	0	5.9–9	5.9–9	0	0
	AZAHAR AND LAVANDER FLAVOURED SOFT DRINK	1	37	0	0	8.8	8.8	0	0.03
	PINK PEPPER SOFT DRINK	1	37	0	0	8.8	8.8	0	0.03
	STRAWBERRY SOFT DRINK STILL, LOW CALORIE	1	12	0.1	0	2.5	2.4	0	0.1
	APPLE SOFT DRINK STILL	1	41	0	0	10	9.9	0	0.1
	APPLE SOFT DRINK STILL, LOW CALORIE	2	9–14	0	0	1.8–3.5	1.8–3.4	0	0–0.1
	TROPICAL SOFT DRINK STILL	1	40	0.1	0	9.3	9.1	0.1	0.1
	PINEAPPLE SOFT DRINK STILL, LOW CALORIE	1	13	0.1	0	2.6	2.4	0	0.1
	MULTIFRUIT SOFT DRINK STILL, LOW CALORIE	2	8–16	0	0	1.4–3.9	1.4–3.9	0–0.1	0–<0.01
	LEMON TEA SOFT DRINK	1	32	0	0	7.7	7.7	0	0.04
	PEACH TEA SOFT DRINK	2	28–32	0	0	6.6–7.8	6.6–7.8	0	0.03–0.05
	LEMON TEA SOFT DRINK LOW CALORIE	1	19	<0.5	<0.1	4.6	4.5	<0.5	0.06
	PEACH TEA SOFT DRINK LOW CALORIE	1	19	<0.5	<0.5	4.7	4.5	<0.5	0.05
	LEMON TEA SOFT DRINK NO-CALORIE	1	1	0	0	0	0	0	0.05
	THEINE-FREE TEA SOFT DRINK, NO-CALORIE	1	1	0	0	0	0	0	0.06
	GREEN TEA SOFT DRINK	2	20	0–<0.5	0–<0.5	4.6–4.7	4.6–4.5	0–<0.5	0.03–0.05
	MANGO AND PINEAPPLE TEA SOFT DRINK	1	34	0	0	8.2	8.1	0	0.04
	GASEOSAS (SODA WATER)	2	0.3–1	0	0	0	0	0	0.03–0.1
	CARBONATED ORANGE DRINK	1	1.5	0	0	0	0	0	0
	CARBONATED LEMON DRINK	1	0.9	0	0	0	0	0	0
	CARBONATED DRINK	2	0	0	0	0	0	0	0.05–0.1
	TONIC WATER	2	36–40	0	0	8.5–9.5	8.4–9.5	0	0–0.03
	GINGER ALE	3	35–38	0	0	8.6–9.1	8.6–9.1	0	0.02–0.05
	TONIC WATER, NO-CALORIE	2	2	0	0	0	0	0	0–0.05
	BITTER	2	32	0	0	8–8.1	8–8.1	0	0–0.03
	BITTER, NO-CALORIE	1	3	0	0	0	0	0	0.01
**Total**		**94**							

Data expressed per 100 mL; SFA: Saturated fatty acids; (-): data not declared.

**Table 3:** Nutrition facts from the “energy drinks” group in the Spanish food market.

Beverages Group	Subgroup	*n*	Energy (kcal)	Fats (g)	SFA (g)	Carbohydrates (g)	Total Sugars (g)	Proteins (g)	Salt (g)	B3 (mg)	B5 (mg)	B6 (mg)	B12 (µg)
**Energy drinks**	ENERGY DRINK	3	45–60	0	0	10.1–14.5	10.1–14.5	0–0.4	0.04–0.28	6.5–8	1.5–2	0.21–2	0.38–2
	LIME ENERGY DRINK	1	45	0	0	11	11	0	0.1	8	2	2	2
	RED FRUITS ENERGY DRINK	1	46	0	0	11	11	0	0.1	8	2	2	2
	CRANBERRY ENERGY DRINK	1	46	0	0	11	11	0	0.1	8	2	2	2
	APPLE ENERGY DRINK	1	46	0.5	<0.1	10.6	10.6	<0.5	0.23	8	2	2	2
	GUARANA ENERGY DRINK	1	45	<1	<1	11.2	11.2	<1	0.28	7.6	2	2	2
	JUICE ENERGY DRINK	1	45	0	0	10.8	10.8	0	0.06	3.8	1	0.21	0.38
	COFFEE FLAVOURED ENERGY DRINK	1	43	0.5	0.4	8.1	8.1	1.3	0.05	3.2	1	0.21	-
	NO-CALORIE ENERGY DRINK	2	2–3	0	0	0	0	0–<0.02	0.05–0.1	6.5–8	1.5–2	0.21–2	0.38–2
**Total**		**12**											

Data expressed per 100 mL; SFA: Saturated fatty acids; (-): data not declared.

**Table 4:** Nutrition facts from the “sports drinks” group in the Spanish food market.

Beverages Group	Subgroup	*n*	Energy (kcal)	Fats (g)	SFA (g)	Carbohydrates (g)	Total Sugars (g)	Proteins (g)	Salt (g)	K (mg)	Ca (mg)	Mg (mg)
**Sports drinks**	LEMON SPORTS DRINK	2	24–31	0	0	5.9–7.5	3.9–7.5	0	0.13	12.5	1.3	0.6
	ORANGE SPORTS DRINK	3	24–31	0	0	5.9–7.5	3,9–7.5	0	0.13	12.5	1.3	0.6–1.6
	RASPBERRY SPORTS DRINK	2	24–31	0	0	5.8–7.5	3.9–7.5	0	0.13	12.5	1.3	0.6
**Total**		**7**										

Data expressed per 100 mL; SFA: Saturated fatty acids.

**Table 5:** Nutrition facts from the “beverages containing mineral salts in their composition” group in the Spanish food market.

Beverages Group	Subgroup	*n*	Energy (kcal)	Fats (g)	SFA (g)	Carbohydrates (g)	Total Sugars (g)	Proteins (g)	Salt (g)	Cl^−^ (mg)	K (mg)	P (mg)	Ca (mg)
	MINERAL SALTS LEMON DRINK	1	26	0	0	6.3	6.3	0	0.05	24	2.2	1	0.8
	MINERAL SALTS ORANGE DRINK	1	32	0	0	7.9	7.9	0	0.05	24	2.2	1	0.8
	MINERAL SALTS ORANGE DRINK, SUGAR-FREE	1	1	0	0	0	0	0	0.05	-	-	-	-
	MINERAL SALTS LEMON DRINK, SUGAR-FREE	1	1	0	0	0	0	0	0.05	-	-	-	-
**Total**		**4**											

Data are expressed per 100 mL; SFA: Saturated fatty acids; (-): Data not declared.

**Table 6:** Nutrition facts from the “vegetable drinks” group in the Spanish food market.

Beverages Group	Subgroup	*n*	Energy (kcal)	Fats (g)	SFA (g)	Carbohydrates (g)	Total Sugars (g)	Proteins (g)	Salt (g)	Ca (mg)
**Vegetable drinks**	SOYA COCOA DRINK	3	53–71	1.6–2	0.3–0.4	6.1–9.4	5.8–9	3–3.4	0.10–0.15	120
	SOYA VANILLA DRINK	3	43–54	1.6–1.9	0.3–0.4	6.1–9.4	5.8–9	3–3.4	0.10–0.15	120
	SOYA ORANGE DRINK	2	46–48	0.3–0.4	0–0.1	9.7–10	9.7–10	0.7–1	0.03–0.1	-
	SOYA PINEAPPLE DRINK	2	47–48	0–0.4	0–0.1	10	9.9–10	0.7–1	0.03–0.1	-
	SOYA PEACH DRINK	2	47–48	0.3–0.4	0–0.1	9.8–10	9.8–10	0.8–1	0.03–0.1	-
	HORCHATA (tiger nut milk)	3	68–78	2–2.3	0.3–0.6	11.5–14.4	10–12.6	0.4–0.6	0–0.2	-
**Total**		**15**								

Data are expressed per 100 mL; SFA: Saturated fatty acids; (-): Data not declared.

**Table 7:** Nutrition facts from the “fruit juice, nectar, and grape musts” group in the Spanish food market.

Beverages Group	Subgroup	*n*	Energy (kcal)	Fats (g)	SFA (g)	Carbohydrates (g)	Total Sugars (g)	Proteins (g)	Salt (g)	C (mg)
**Fruit juice, nectar, and grape musts**	ORANGE JUICE	4	43–46	0–<0.5	0–<0.1	9–10.5	8.1–10	0	0.001–0.03	10
	PINEAPPLE JUICE	3	47–55	0–0.1	0	11.2–13.2	11.2–12.1	0.3–0.6	0.002–0.01	6
	PEACH JUICE	2	42–53	0–0.1	0	10.1–11.9	10.1–11.9	0.4–0.8	0.002–0.01	-
	APPLE JUICE	3	41–51	0–0.2	0–0.1	10–12	9.7–11.7	0	0–0.01	-
	TOMATO JUICE	2	14–20	0–0.2	0–0.1	2.6–4.8	2.6–4.8	0–1	0–0.29	-
	MULTIFRUIT JUICE	4	50–58	0–0.2	0–0.1	11.3–13.5	9–13.5	0.5–0.8	0–0.025	-
	ORANGE NECTAR	3	45–46	0–0.2	0–0.1	10.3–11.2	10.3–11.2	0–0.5	0–0.01	32
	ORANGE NECTAR, NO ADDED SUGARS	4	19.6–33	0–0.2	0–0.1	4.6–7.6	4–7.6]	0–0.5	0–0.03	-
	PINEAPPLE NECTAR	3	48–53	0–0.2	0–0.1	11.8–12.5	11.8–12.5	0–0.5	0–0.03	-
	PINEAPPLE NECTAR NO ADDED SUGARS	3	23–36	0–0.2	0–0.1	5.4–8.4	5.4–8.4	0–0.5	0–0.03	-
	PEACH NECTAR	3	46–59	0–0.5	0–0.1	11.3–14.3	11.3–14.3	0–0.5	0–0.01	-
	PEACH NECTAR, NO ADDED SUGARS	3	18–32	0–0.2	0–0.1	4.2–7	3.8–6.9	0.3–0.5	0–0.03	-
	MULTIFRUIT NECTAR	1	45	0	0	11.1	11.1	0	0	-
	MULTIFRUIT NECTAR NO ADDED SUGARS	2	24–31	0.1–0.2	0–0.1	5.4–7	4.2–7	0.2–0.5	0–0.03	-
	MEDITERRANEAN SMOOTHIES	3	29–48	0	0	6.6–11.3	6.6–11.3	0–0.4	0.02–0.06	12
	TROPICAL SMOOTHIES	3	21–53	0	0	4.8–12.6	4.8–12	0–0.4	0.02–0.1	12
	GRAPE MUSTS	3	48–69	0	0	12–17	12–17	0.1–0.2	0–0.01	-
**Total**		**49**								

Data are expressed per 100 mL; SFA: Saturated fatty acids; (-): Data not declared.

**Table 8:** Nutrition facts from the “dairy drinks” group in the Spanish food market.

Beverages Group	Subgroup	*n*	Energy (Kcal)	Fats (g)	SFA (g)	Carbohydrates (g)	Total Sugars (g)	Proteins (g)	Salt (g)
**Dairy drinks**	STRAWBERRY LIQUID YOGURT	5	69–88	0.7–2.5	0.4–1.5	12.7–14	12.3–14	2.4–3	0.1
	STRAWBERRY AND BANANA LIQUID YOGURT	3	69–86	0.7–2.2	0.4–1.4	12.7–14	12.3–14	2.4–3	0.1
	NATURAL LIQUID YOGURT	3	76–84	1.1–2.6	0.7–1.6	11.6–14	11.5–14	2.8–3.1	0–0.1
	SKIMMED LIQUID YOGURT	3	33–36	0.2–0.4	0.1–0.2	3.6–5.7	3.5–4.6	2.6–3	0.10–0.13
	COCOA MILKSHAKE	5	64–80	0.6–1.6	0.4–1.1	10–13.8	10–13.4	2.1–3.3	0.12–0.17
	VANILLA MILKSHAKE	3	60–68	0.4–1	0.3–0.6	10–13	10–13	2.6–3.1	0.13–0.15
	STRAWBERRY MILKSHAKE	3	64–67	0.4–1	0.3–0.6	11–13	11–13	2.6–3.1	0.13–0.15
	COFFEE DRINK	4	59–80	1.2–4	0.7–2.4	8–10	8–10	3	0.15
	COFFEE DRINK, LOW CALORIE	1	39	0.8	0.5	5.5	5.5	2.5	0.15
**Total**		**30**							

Data are expressed per 100 mL; SFA: Saturated fatty acids.

**Table 9:** Classification of non-alcoholic beverages in the Spanish Food Composition Tables.

Food Composition Table or Database	Drinks Groups	*n*
BEDCA network [1]	**Non-dairy:**	
	Soft drinks	14
	Juices	14
	Nectars	10
	Horchatas	1
	**Dairy and milk derivatives:**	
	Milkshakes	4
	Soya shake	2
	Soya drink	1
	Liquid yogurt	13
Moreiras, O. et al. (2015) [4]	**Milk and milk derivatives:**	
	Milkshakes	4
	Liquid yogurt	3
	**Non-alcoholic beverages:**	
	Soya shake	1
	Soft drinks	9
	Horchata	1
	Juices and nectars	15
Ortega, et al. (2008) [7]	**Dairy and milk derivatives:**	
	Yogurt and fermented milks	3
	Milkshakes	3
	**Beverages:**	
	**Non-alcoholic beverages:**	
	Soft drinks	11
	Commercial juices and nectars	9
	Other non-alcoholic beverages: horchata	1
CESNID (2004) [5]	**Milk and milk derivatives:**	
	Milks and milk shakes	5
	**Non-alcoholic beverages:**	
	Soft drinks	9
	Packaged juices and nectars	17
	Other non-alcoholic beverages: horchata, isotonic drink	2
Mataix, J. (2011) [6]	Fruit juices	22
	Milk and milk derivatives	18
	Drinks	6
